# The National Association of Railway Surgeons

**Published:** 1891-06

**Authors:** 


THE NATIONAL ASSOCIATION OF RAILWAY SURGEONS held a two days' meeting in Buffalo, commencing April 30th. About 150 members were present from various parts of the Union, chiefly from the south and west. A number of interesting papers were read and discussed. Dr. and Mrs. B. H. Daggett, of this city, gave a reception in honor of the delegates on Thursday evening, April 30th, which was largely attended and was a most enjoyable occasion. An excursion was given to the members to Niagara Falls, on Saturday, May 2d, after the final adjournment. Dr. Murphy, of St. Paul, was chosen President, and the next place of meeting is White Sulphur Springs, W. Va.

